# Use of Sports Supplements in Competitive Handball Players: Sex and Competitive Level Differences

**DOI:** 10.3390/nu12113357

**Published:** 2020-10-31

**Authors:** Alejandro Muñoz, Álvaro López-Samanes, Raúl Domínguez, Víctor Moreno-Pérez, Antonio Jesús Sánchez-Oliver, Juan Del Coso

**Affiliations:** 1 Exercise Physiology Group, Exercise and Sport Sciences, Faculty of Health Sciences, Universidad Francisco de Vitoria, 28223 Madrid, Spain; alejandro.muñoz@ufv.es; 2 Faculty of Physical Activity and Sports Sciences (INEF), Universidad Politécnica de Madrid (UPM), 28040 Madrid, Spain; 3 Exercise Physiology Group, School of Physiotherapy, Faculty of Health Sciences, Universidad Francisco de Vitoria, 28223 Madrid, Spain; 4 Studies Research Group in Neuromuscular Responses (GEPREN), University of Lavras, 37200-000 Lavras, Brazil; raul_dominguez_herrera@hotmail.com; 5 Escuela Universitaria de Osuna (Attached Universidad de Sevilla), 41640 Sevilla, Spain; 6 Departamento de Educación Física y Deporte, Universidad de Sevilla, 41013 Sevilla, Spain; 7 Sports Research Centre, Miguel Hernández University of Elche, 03202 Alicante, Spain; vmoreno@goumh.umh.es; 8 Departamento de Motricidad Humana y Rendimiento Deportivo, Universidad de Sevilla, 41013 Sevilla, Spain; sanchezoliver@us.es; 9 Centre for Sport Studies, Rey Juan Carlos University, 28943 Fuenlabrada, Spain; juan.delcoso@urjc.es

**Keywords:** nutrition, team sports, intermittent sports, ergogenic aids, elite athlete

## Abstract

Sports supplements are commonly used by elite athletes with the main goal of enhancing sport performance. Supplements use might be substantially different depending on the sport discipline, sex, and competitive level. To date, data about prevalence and the most-commonly used supplements in handball are scarce. Thus, the aim of this investigation was to determine the patterns of supplements use by handball players of both sexes and with different competitive levels: One hundred and eighty-seven handball players (112 men and 75 women) of different competitive levels (106 professional and 81 amateur) completed a validated self-administered questionnaire about supplements use. Supplements were classified according to the categorization of the Australian Institute of Sport (AIS). Overall, 59.9% of the handball players (*n* = 112) declared the use of at least one supplement and there were no significant differences between men and women (58.9% vs. 61.3%, *p* = 0.762) nor between professional vs. amateur handball players (67.1% vs. 53.8%, *p* = 0.074). The most prevalent supplements were sports drinks (42.2%), followed by energy bars (35.3%) and caffeine-containing products (31.6%). However, a greater consumption of group A supplements (those with strong scientific evidence; *p* = 0.029) and group B supplements (those with emerging scientific support, *p* = 0.012) was observed in male compared to female handball players. Supplements categorized as medical supplements were more commonly consumed in professional vs. amateur players (0.48 ± 0.80 vs. 0.21 ± 0.44, supplements *p* < 0.006). Additionally, a higher consumption of group B supplements was observed in professional compared to amateur players (0.58 ± 0.88 vs. 0.33 ± 0.72 supplements, *p* = 0.015). Handball players revealed a moderate use of supplements while sex and competitive level slighted changed the pattern of supplements use. A high portion of handball players use supplements as fuel during exercise and reported the use of caffeine-containing supplements to enhance performance.

## 1. Introduction

Handball is an intermittent team sport that combines short bouts of high intensity exercise interspersed with periods of moderate-to-low exercise intensity [1]. Although the physical demands of handball might depend on the on-court playing position [2], all-out actions such as accelerations, decelerations, changes-of-direction, sprints, jumps, and shots is a common denominator of handball performance [3]. The unlimited number of substitutions permits time for recovery off the court, which in turn increases the intensity of in-court actions, and at the elite level, it allows distinct attack/defense roles for some players. Another handball-specific characteristic is the allowance of body contact, as defenders may tackle and block attackers when shooting while the attacking handball players continuously struggle with defenders to create openings for teammates or to get good scoring positions for themselves [4]. Unlike other team sports which penalize body contact, this handball feature implies the necessity of high levels of muscle strength where players’ body mass might be considered as a performance factor for some playing positions [5]. The relevance of body contact for sport performance is shared with rugby and it may lead handball players to use of dietary supplements with potential benefits associated to lean body mass gain, such as creatine [6,7]. Overall, competitive handball players need a mixture of technical, tactical, physical, and psychological aspects to produce an excellent performance during matches [8]. Thus, physical aptitude in handball has been related to high values of strength and power output in both upper and lower body, together with agility and high accelerative and jumping capacities [1]. This large number of high-intensity efforts during a handball game has attracted the attention of sports scientists who are looking for strategies to increase performance through enhanced recovery [9] or nutritional measures [10,11]. The use of dietary supplements is a potentially valuable strategy to achieve success in professional handball where players have a demanding calendar with national and international competitions. To this regard, the use of dietary and sports supplements to achieve a specific and direct performance benefit during game play, to allow more effective training and better recovery between training sessions, or to reduce the risks of injury and illness, may be beneficial for overall handball performance.

Sports supplements can be defined as a food, food component, or nutrient that is purposefully ingested, in addition to the habitually consumed diet, with the aim of achieving a specific physical performance or health benefit [12]. Supplements are commonly used by endurance and intermittent sports athletes (e.g., athletics, cycling, soccer, tennis, basketball) during training/competitions [13,14,15,16], although the reasons for use might be different depending on the characteristics of the sport [17]. There is substantial consumption of supplements at all competitive levels, although, in general, elite athletes use more dietary supplements than their non-elite counterparts [17,18,19,20]. With few exceptions, the prevalence in the use of supplements is similar for men and women [21], although men athletes tend to consume more protein-based supplements, and women athletes tend to use more iron supplements [22], potentially due to the greater prevalence of iron deficiency in females [23,24]. One of the most recurrent result in the investigations about the use of supplements in sport is the existence of notable differences in the amount and type of supplements used among sports [18,21]. The differences in the pattern of supplements use among sports are likely linked to differences in the physical and physiological determinants for success in each discipline, together with variations in the idiosyncrasy of each sport discipline. Overall, it seems that athletes use a high proportion of supplements with low level of evidence [18], likely because a high portion of athletes use unreliable sources of information (i.e., they rely on themselves for the obtaining of valid and accurate information about the efficacy of dietary supplements and they are the main responsible for the plan of supplementation [25]). Additionally, the lack of guidance from qualified professionals when planning the use of dietary supplements is more common in men than in women [26]. Interestingly, it has been found that receiving dietary counseling by a qualified professional, instead of self-prescription of supplements, results in better-informed choices with respect to the use of nutritional supplements [27]. These circumstances, added to the high proportion of athletes purchasing supplements on the internet [18], increase the risks of supplements misuse, or the risks of inadvertent doping, particularly in men. Specifically, the benefits of a higher body mass and enhanced muscle power and strength in handball may be associated with inappropriate supplementation practices and with the use of some prohibited substances that allow fast increases in body mass and reductions in body fat (e.g., anabolic androgenic steroids), as it happens in rugby [6,7].

The use of supplements in endurance sports has been extensively studied [27], while knowledge about patterns of supplements use in team sports, particularly handball, is very limited. Interestingly, handball players seem to have a higher knowledge about sports nutrition and dietary supplements than basketball, football, and volleyball players, although only 15.5% of handball players reported consumption of dietary supplements regularly [28]. Last, few studies have been carried out to assess the efficacy of evidence-based supplement in handball, such as creatine [29,30], caffeine [31,32], citrulline/malate [33], and beetroot juice ingestion [34], in line with accepted professional guidelines, such as the Australian Institute of Sport (AIS) supplement framework.

To our knowledge, only one previous study has analyzed the use of supplements in handball players [28]. In this study, with a sample of 206 professional team handball players, the authors observed that 49% of the players consumed at least one supplement. However, Sekulic et al. [28] did not investigate differences by sex or competitive level in the use of dietary supplements. Thus, the aim of this study was to analyze the use of supplements in handball players by describing the pattern of consumption as a function of sex and competitive level (professional vs. amateur).

## 2. Materials and Methods

### 2.1. Participants

A total of 187 players (112 men and 75 women) participated in this investigation. The sample was divided into two groups: one group composed of 106 professional players (i.e., professionals handball players that compete in the First Division of the Spanish Handball League (ASOBAL)) and the second group, composed of 81 amateur players (Spanish Second and Third Divisions). Participants were recruited with the help of the Spanish Handball Federation which distributed an email with specific instructions and the online version of the questionnaire to all the men’s and women’s First Division Teams of ASOBAL, and to other non-professional handball teams. Potential participants also distributed the email with the questionnaire among teammates and thus, it was unfeasible to record the exact number of athletes solicited for this investigation. From the total of questionnaires received (189), 1.06% were removed because they were incomplete, or had been incorrectly filled out, for a total of 187 valid questionnaires for analysis. This research study was approved by the Ethics Committee of Alfonso X El Sabio University and complied with the Declaration of Helsinki.

### 2.2. Experimental Design

All handball players voluntarily participated in this investigation and they were only required to complete a self-report questionnaire about their use of supplements. The questionnaire used in this investigation has been previously validated to assess the prevalence and patterns of supplements use by athletes [35] and it has been used in previous investigation with this aim [20,36,37]. Briefly, the questionnaire was developed by a group of three experienced sport scientists and its construct validity was verified by a group of 25 experts in nutrition, sports sciences, sports medicine, pharmacology, and chemistry. The questionnaire contained a clear definition of a supplement according to the AIS [38], depicted several examples to differentiate the different types of supplements, and contained information to contact the researchers in the case that participants required direct communication to solve any doubt regarding the questions included in the questionnaire. Participants filled out the questionnaire between August 2018 and October 2019 and it was prepared in a web-based form (Google Forms, Google, Mountain View, CA, USA) to allow distribution and recollection of information. For the data collection, participants received an online version of the questionnaire to thoughtfully record their use of supplements, along with demographic and training information to correctly catalogue the sample. This questionnaire specifically asked about the number of supplements consumed at the moment of filling out the questionnaire, the aim and expectancies of the supplements consumption, the sources of information consulted to use the supplement, and the habitual place of purchase for the supplements. The information collected by the questionnaire does not allow the identification of the players to reduce the potential bias in the responses due to the feeling of lack of anonymity. In addition, the questionnaire also had a section to be filled out only by those who did not report any supplements use. In a review conducted by Knapik et al. [21] that assessed the quality of questionnaires aiming to determine the prevalence in the use of dietary supplements by athletes, this same questionnaire was rated as presented in a publication by Sanchez-Oliver et al. [36] and it achieved 54% methodological quality. Briefly, the methodological quality in the Knapik’s evaluation was rated by using an 8-point scale that included assessments for sampling methods, sampling frame, sample size, measurement tools, bias, response rate, statistical presentation, and description of the participant sample. The percentage of methodological quality was obtained by the ratings obtained in all these characteristics of the questionnaire and the current questionnaire was one of the 57 questionnaires (out of 164) reviewed that were considered suitable to obtain accurate information of supplements use by athletes.

### 2.3. Statistical Analysis

Data are presented as means and standard deviation (SD) for the participant characteristics, whereas frequencies and percentages have been used for the remaining variables. The Kolmogorov-Smirnov test was used to assess the normal distribution of the data, while homoscedasticity was obtained with Levene’s test. Data were organized by sex (men vs. women) and competitive level (professional vs. amateur). Comparisons for the continuous variables such as number of supplements used, were analyzed using two-way ANOVA (level × sex). The Bonferroni post-hoc test was used to detect pairwise differences in the case of a significant *p* value. Chi-square tests (χ2) were applied to identify differences in categorical variables (e.g., motivation, expectations, and contextualization of the use of supplements). Once participants had filled out the questionnaire, their responses regarding the type of supplements used were categorized according to the ABCD system of scientific evidence established by the Australian Institute of Sport (AIS) [37]. Additionally, in those supplements in which prevalence in consumption was greater than 10% of the sample, a χ2 test was performed to detect possible differences both as a function of the participants’ sex and competitive level. Statistical significance was established at *p* < 0.05. The statistical analysis was performed using the Statistical Package for Social Sciences (version 18.0 for Mac, SPSSTM Inc, Chicago, IL, USA).

## 3. Results

**Figure 1:**
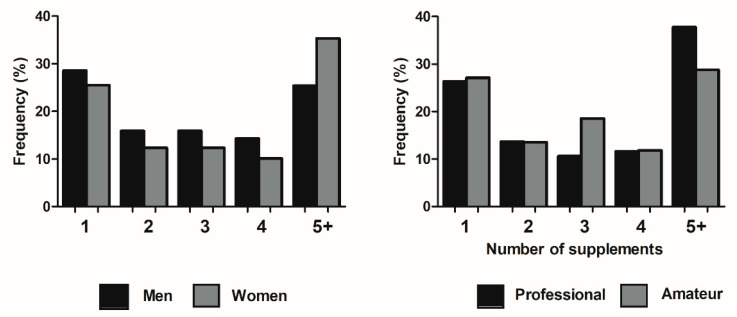
Number of supplements consumed according to sex (left panel) and competitive level (right panel). Data are mean ± standard deviation for 112 players who reported the use of at least one supplement in the questionnaire.

Table 1 shows descriptive values of body characteristics, experience, and training volume. Overall, 59.9% of the study sample reported the use of at least one supplement at the moment of filling out the questionnaire. There was no significant difference in the prevalence of supplements use between male and female handball players (58.9% vs. 61.3%, respectively; *p* = 0.762), nor between amateur vs. professional handball players (53.8% vs. 67.1%; *p* = 0.074). In addition, no significant differences were found in the number of supplements used in men compared to women (2.69 ± 2.47 vs. 1.97 ± 2.19, *p* = 0.052). However, professional players reported the use of a higher number of supplements than amateur players (2.73 ± 2.38 vs. 2.19 ± 2.32; *p* = 0.033). There were no significant differences in the frequency distribution in men vs. women (*p* = 0.217) nor in professional vs. amateur (*p* = 0.098) according to the number of supplements used (Figure 1).

**Figure 2:**
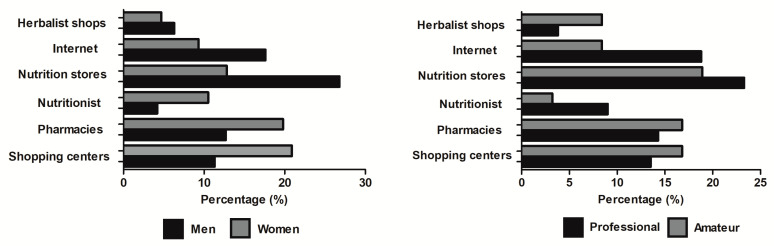
Main site of purchase of sports supplements in handball players according to sex (left panel) and competitive level (right panel).

Overall, the most frequent reasons for supplements use were enhancing sports performance (54.1%), improving health (12.7%), and improving physical appearance (10.9%) with no sex differences in the frequency of the reasons to use supplements (*p* = 0.146). In the group handball players that reported the use of at least one supplement (*n* = 112), the likelihood of this supplement being used to enhance sports performance was 4.25-fold higher (*p* < 0.001) than being used for improving health, and 4.96-fold higher (*p* < 0.001) than for improving physical appearance. The frequency of professional players that reported the use of supplements to enhance performance (51.9%) was higher than the frequency in amateurs that used supplements with this aim (34.0%; *p* = 0.026). According to the purchase sites, statistical differences were reported by sex (*p* = 0.021) and by competitive level (*p* = 0.020; Figure 2). Specifically, male handball players mainly purchased supplements in specialized nutrition stores while female players primarily purchased supplements in non-specialized shopping centers.

**Figure 3:**
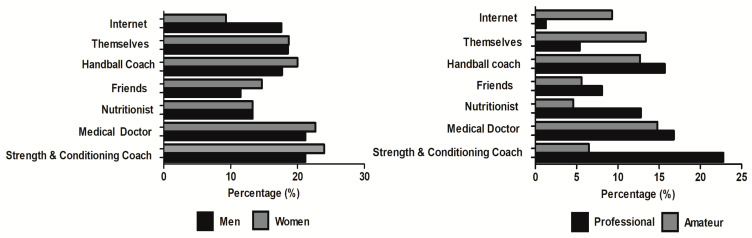
Sources of information when planning to use dietary supplementation in handball players according to sex (left panel) and competitive level (right panel).

Concerning where players sought information to decide on the use of supplements, both men and women handball players mainly consulted their strength and conditioning coach followed by the team doctor, with no significant differences between sex (*p* = 0.109). The reliance on the strength and conditioning coach was higher in professional than in amateur players while a higher reliance on themselves and on internet was found in amateur players (*p* < 0.001; Figure 3).

Table 2 contains information about the number of supplements according to the categories proposed by the AIS. Regarding the number of sport foods, no significant differences were observed by either competitive level or sex (Table 2). Regarding the number of medical supplements, a higher consumption was observed in professional handball players when compared to amateur counterparts (0.48 ± 0.80 vs. 0.21 ± 0.44 supplements, *p* < 0.006). Regarding the category sport performance supplements, a greater consumption of group A (0.63 ± 0.70 vs. 0.37 ± 0.09 supplements, *p* = 0.029) and group B (0.59 ± 0.08 vs. 0.27 ± 0.10 supplements, *p* = 0.012) supplements was reported in men than in women handball players. In the category of group B supplements, professional handball players also consumed more supplements than amateur players (0.58 ± 0.88 vs. 0.33 ± 0.72 supplements, *p* = 0.015). Finally, low and equal consumption of group C supplements was reported in all categories of handball players.

**Figure 4:**
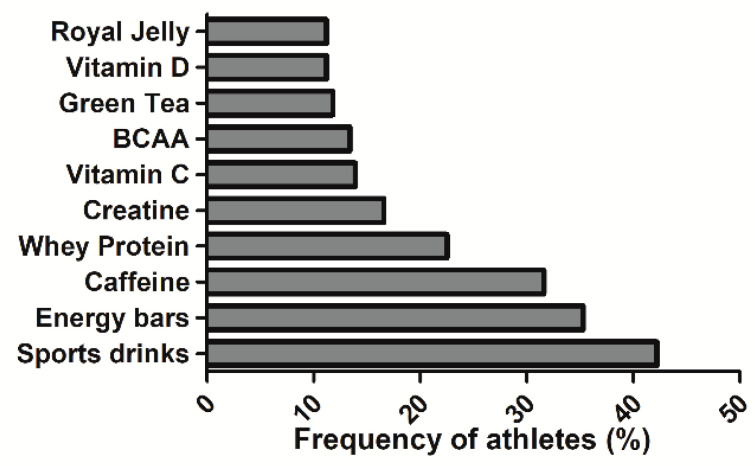
Type of sports supplements most commonly used in handball players. Frequency refers to the percentage of players that used each type of sport supplement with respect to the number of participants who reported use of sports supplements. The sum of all percentages is > 100% because there were players that used more than one sport supplement. BCAA: Branched Chain Amino Acids.

Table 3 and Table 4 show the fourteen supplements in which the overall prevalence of consumption was greater than 10%. The most consumed supplement was sports drinks, followed by energy bars and caffeine-containing products (Figure 4). When comparing the consumption rates according to the competitive level, it was found that professional handball players had a higher consumption of vitamin D, vitamin complexes, and creatine than amateurs (all *p* < 0.050). Regarding sex, a higher consumption was observed in male handball players in terms of creatine and L-carnitine than in female handball players (all *p* < 0.050).

## 4. Discussions

The aim of this study was to determine the prevalence of supplements use in professional and amateur handball players to characterize the use of supplements in handball according to sex and competitive level. Previously, Sekulic et al. [28] reported that 49% of professional handball players used at least one supplements, a prevalence similar to the overall prevalence obtained in the current study (59.9%). However, the current study is innovative because it reflects that the prevalence in the use of supplements is not affected by the competitive level (professional vs. amateurs) or by sex (men vs. women). In comparison with other intermittent team sports, the prevalence in the use of supplements in handball players was similar to basketball players (53–56.1%) [15,28], soccer players (50–58%) [28,39,40], and volleyball players (46% users) [28]. However, the prevalence of supplements in handball was lower than that found in racket sports such as tennis (81.7%) [14], and squash (81.0%) [20]. Most of the literature reports that professionals athletes have a higher prevalence in the use of supplements and they usually consume a higher number of supplements than their counterparts of a lower competitive level [14,41], although this is not always the case [42]. In this regard, professional handball players used a higher number of supplements than their amateur counterparts. However, contrary to previous literature [18,21,28,43], the differences in the number of supplements consumed by male and female handball players did not reach significance. Interestingly, the proportion of women reporting the consumption of five or more supplements was higher than in men and in professional players than in amateur handball players (Figure 1). Together, all this information suggests that handball players had an overall moderate use of supplements which is comparable to other team sports. Professional handball players reported the use of a higher number of supplements than amateur handball players, but the prevalence in the use of supplements was not affected by the sex of the player. It may be important to highlight that the data obtained in this study refers to supplement consumption throughout the season and it does not reflect intermittent changes in this pattern that may occur at different stages during the handball season.

The Australian Institute of Sport has proposed an ABCD categorization system to catalogue each supplement according to the existing level of scientific evidence regarding its effectiveness to fulfil a performance or health benefit [38]. By using this categorization, we have found that professional men handball players reported a higher number of group-A medical supplements aimed to increase performance than their amateur counterparts. There was also greater consumption by professional male handball players compared to professional female handball players in the category of sports performance supplements (included in group A) and in the group-B supplements. Finally, no significant differences were detected depending on the level of players in the supplements of group C [20]. As a whole, these data might indicate that handball players mostly used supplements with a good level of scientific evidence, particularly professional male and female players. On the contrary, the percentage of players that used supplements with a low level of scientific evidence was always lower than 15%.

Overall, the most frequent reason to use dietary supplements in this sample of competitive handball players was the intention of obtaining a performance benefit, which was several-fold higher to the use of supplements to enhance health or for aesthetic reasons. Sousa et al. [41], in professional Portuguese athletes, and López-Samanes et al. [14], in professional tennis players, also found that the most important reason for supplements use was to obtain a performance enhancement. Thus, it seems that, although the market of supplements is diverse in products and in purported benefits, the most common approach of athletes to the use of supplements in a sporting context is to obtain a benefit that may impact their sports performance. Although there were no significant differences, the main reason for men handball players to use supplements was to obtain a sports performance enhancement while the most frequent reason in women handball players was to obtain a health benefit. A similar tendency has been previously reported in samples of elite athletes [14,37,41] which might support the idea that, despite having a similar prevalence in the use of dietary supplements, the reasons for using supplements are slightly different between male and female athletes, with females normally more concerned about supplements that produce a health benefit [26]. Interestingly, a higher proportion of professional handball players used supplements to increase performance (51.9%) than amateur counterparts (34.0%). All this information suggests that, in handball, the obtaining of a performance benefit is the main reason for the use of supplements while the reported motivations for supplementation use may be modified by sex and competitive level.

In relation to the site of purchase, subtle differences were observed depending on the competitive level of the players. The specialized nutrition stores were the preferred place of purchase for professionals and these also were the most common sites of purchase in amateur handball players [44]. However, it is noteworthy to mention the high frequency of professional handball players that purchased supplements on the Internet, despite the information that points toward a high prevalence of low-quality or tampered supplements due to the absence of specific legislation [45]. Although the online market of dietary and sports supplements has made easier the purchase of these products on the Internet, some authors believe that this constitutes a public health problem due to the high percentage of supplements available on the Internet with prohibited or unlabeled substances [46]. To this regard, contamination in a supplement can occur through sub-standard manufacturing, leading to cross-contamination of products with a prohibited substance during the production of the supplement itself [47]. In addition, intentional contamination may also occur when the unlabeled/prohibited substance is added to the supplement with the aim of enhancing its effects [48]. The free sale of these products on the Internet, sometimes without the control of countries’ health authorities [45], likely increases the probability of purchasing a supplement of lower quality or with prohibited substances. All this information indicates that a high frequency of handball players may be at risk of inadvertent doping due to their behaviors when purchasing supplements. It is important to mention that inadvertent doping is not an extenuating circumstance when an anti-doping rule violation is committed as The Code of the World Anti-Doping Agency clearly indicates that one of the athlete’s responsibilities is to ensure that no prohibited substance enters his or her body. Additionally, it is not necessary that intent, fault, negligence, or knowing use on the athlete’s part be demonstrated to establish an anti-doping rule violation under the circumstances described in The Code [49]. At the same time, a high proportion of professional handball players relied on the opinions of their strength and conditioning coaches to manage the use of supplements while professionals such as medical doctors or nutritionists were less frequently consulted. Moreover, a considerable proportion of amateur handball players relied on themselves or on the information obtained through internet sources to plan their supplements use. These outcomes may indicate that some professional and amateur handball players have inadequate behaviors when using supplements, particularly when purchasing supplements and when obtaining information about supplements. It is important to highlight that these inadequate behaviors do not only entail the use of low-level of evidence supplements in handball, but they may involve other negative effects with legal consequences. For this reason, specific policies to inform about best guidelines to search for information and to purchase supplements should be given to professional handball players, at least in Spain.

In handball players, the most-consumed supplements were sports drinks followed by energy bars and caffeine-containing products. The use of these supplements was high irrespective of players’ sex and competitive level (Table 3 and Table 4). Sports drinks and energy bars are within the most popular supplements in different intermittent sports such as squash and tennis [14,20,28]. The high use of supplements that provide energy is likely because they provide macronutrients, especially carbohydrates, a more convenient form than normal foods for general nutrition support [12] Regarding caffeine, data on urinary caffeine concentration indicates that handball is within the team sports with the highest use of caffeine before sports competition [50]. In addition, there is new evidence showing the ergogenic effect of caffeine to increase several aspects of handball performance [32]. In this case, caffeine was consumed by one third of the sample of handball players, with a similar proportion in professional and amateur players and between male and female handball players. As a whole, the most-consumed sports supplements in handball indicate a clear intention of the players to use exogenous carbohydrates as fuel during exercise and, to use caffeine to obtain the benefits of this substance on several aspects of handball performance. Special mention should be made to creatine-containing supplements. There was a higher use of these supplements in men compared to women, and in professional compared to amateur handball players. Although creatine supplementation has reported different health and sport performance benefits, it is primarily used to gain muscle strength and muscle mass, and to enhance the rate of phosphocreatine resynthesis during recovery periods when training and competing [51]. These effects are of great importance in competitive handball as body contact is permitted to defend against the attackers. The tendency for a higher use of creatine and protein in men professional handball may be related to the higher importance of muscle strength and muscle mass in this category. However, further investigations may be needed to determine the use of other non-legal supplements in handball as there is a high risk of inappropriate supplementation and doping in those sports where body mass, muscle mass, and muscle power/strength are key variables of performance [52].

The current investigation has several limitations that should be discussed to correctly apply the outcomes in competitive handball. First, we used a validated and reliable questionnaire to assess the use of dietary supplements in elite athletes [35]. However, this tool collects self-reported information in a retrospective manner, which might have induced some error due to imprecision in the number and type of supplements reported. In addition, the questionnaire was collected once during the season which precludes the obtaining of in-season variations in the number and type of supplements used. Second, although the questionnaire did not collect any information that permitted the identification of the handball player, it is possible that some athletes may have intentionally avoided reporting some information regarding supplement consumption. This might have affected the proportion of athletes that recognized the use of dietary supplements, with the presence of false negatives (athletes who used supplements but did not report supplement use due to some bias), particularly in the subgroup of professional handball players. In addition, some athletes showed some difficulties to describe the type of supplement they were using when completing the questionnaire. Although the questionnaire contained examples to aid in the identification of supplements, there is still the possibility that some players incorrectly reported the dietary supplements they were actually using. Finally, a lack of questions on doping regulations/screening should be considered in future investigations as this information may be essential to know how the likelihood of inadvertent doping affects the purchase on supplements in elite and amateur athletes.

## 5. Conclusions

Overall, handball players reported a moderate use of supplements and there were no significant differences in the prevalence of use between men and women nor between professional vs. amateur handball players. As a whole group, handball players mainly consumed sports drinks, energy bars, and caffeine with the aim of increasing performance. However, sex and competitive level affected some patterns of use and the most-consumed supplements in handball. A higher proportion of male handball players used creatine while female handball players relied more on non-specialized shops to purchase supplements. Professional handball players used a higher number of supplements, primarily consulted their strength and conditioning coach as a source of information when planning to use sport supplementation, and nutrition stores were the most common place of purchase. On the contrary, a high proportion of amateur handball players relied on themselves to plan supplements use. The relatively low use of supplements with a low scientific level of efficacy suggests that this sample of handball players was well informed about the most effective supplements in their sports.

## Figures and Tables

**Table 1:** Participants characteristics.

Variable	Sex	Professional	Amateur
Height (m)	Men	1.81 ± 0.37 ^λ^	1.79 ± 0.07 ^λ^
	Women	1.64 ± 0.33	1.68 ± 0.07
	Total	1.72 ± 0.36	1.76 ± 0.08
Body mass (kg)	Men	90.08 ±13.37 *^,λ^	81.42 ± 12.09 ^λ^
	Women	65.81 ± 8.51 *	61.48 ± 8.85
	Total	77.94 ± 16.52 *	76.25 ± 14.31
Experience (year)	Men	9.38 ± 1.54 ^λ^	9.20 ± 1.75 ^λ^
	Women	8.40 ± 2.10 *	6.68 ± 3.60
	Total	8.89 ± 1.89 *	8.52 ± 2.50
Training volume (sessions/week)	Men	5.04 ± 1.14 *	2.82 ± 1.16
	Women	4.89 ± 1.20 *	3.05 ± 1.21
	Total	4.96 ± 1.17 *	2.88 ± 1.17

Data expressed mean ± standard deviation (SD) for 112 men and 75 women (106 professional players and 81 amateur players). From this sample, 52 were male professional players, 53 were female professional players, 60 were male amateur players, and 22 were female amateur players. * Difference between professional and amateur at *p* < 0.05; *λ* Difference between men and women at *p* < 0.05.

**Table 2:** Number of supplements used in handball players depending on sex and competitive level.

					*p* Value	
Type of Supplement		Sex	Professional	Amateur	Level	Sex
Group A	Sport foods	Men	1.00 ± 1.02	1.08 ± 1.04	0.491	0.686
		Women	1.13 ± 1.17	0.82 ± 0.85		
		Total	1.06 ± 1.09	1.01 ± 0.99		
	Medical supplements	Men	0.53 ± 0.89 *	0.24 ± 0.47	0.006	0.356
		Women	0.43 ± 0.69	0.14 ± 0.35		
		Total	0.48 ± 0.80 *	0.21 ± 0.44		
	Sport Performance	Men	0.70 ± 0.77 ^λ^	0.56 ± 0.82	0.149	0.029
		Women	0.47 ± 0.70	0.27 ± 0.46		
		Total	0.58 ± 0.74	0.48 ± 0.74		
Group B		Men	0.77 ± 1.01 ^λ^	0.41 ± 0.81	0.015	0.012
		Women	0.40 ± 0.69	0.14 ± 0.35		
		Total	0.58 ± 0.88 *	0.33 ± 0.72		
Group C		Men	0.00 ± 0.00	0.20 ± 0.85	0.698	0.698
		Women	0.13 ± 0.62	0.00 ± 0.00		
		Total	0.07 ± 0.44	0.15 ± 0.73		

Data expressed mean ± standard deviation (SD) for 112 men and 75 women (106 professional players and 81 amateur players). Group A: Supplements with strong scientific evidence for use in specific situations in sport using evidence-based protocols; Group B: supplements with emerging scientific support, deserving of further research; Group C: supplements with scientific evidence not supportive of benefit amongst athletes. * Significant difference between professional and amateur; *λ* significant difference between men and women; Statistical significance at *p* < 0.05.

**Table 3:** Most-commonly used supplements according to sex in a sample of handball players.

Type of Supplement		Supplement	Sex		
			Men	Women	*p* Value
Group A	Sport foods	Sports Drinks	42.0%	42.7%	1.000
		Energy Bars	33.0%	38.7%	0.440
		Whey protein	25.9%	17.3%	0.115
	Medical Supplement	Vitamin D	12.5%	9.3%	0.638
		Iron	8.0%	14.7%	0.157
		Vitamin complex	12.5%	8.0%	0.470
	Sport Performance	Caffeine	33.0%	29.3%	0.633
		Creatine	22.3%	8.0%	0.010 *
Group B		Vitamin C	13.4%	14.7%	0.832
		BCAA	16.1%	9.3%	0.273
		L-Carnitine	17.0%	0.0%	<0.001 *
Group C		Green Tea	10.7%	13.3%	0.646
		Royal Jelly	11.6%	10.7%	1.000
		Glutamine	14.3%	5.3%	0.057

Data are frequencies for 112 men and 75 women (106 professional players and 81 amateur players). * Statistical difference in the frequency of consumption between groups (*p* < 0.05). Group A: Supplements with strong scientific evidence for use in specific situations in sport using evidence-based protocols; Group B: supplements with emerging scientific support, deserving of further research; Group C: supplements with scientific evidence not supportive of benefit amongst athlete. BCAA: Branched Chain Amino Acids.

**Table 4:** Most-commonly used supplements according to competitive level in a sample of handball players.

Type of Supplement		Supplement	Level		
			Professional	Amateur	*p* Value
Group A	Sport foods	Sports Drinks	39.6%	45.7%	0.456
		Energy Bars	34.0%	37.0%	0.758
		Whey protein	27.4%	16.0%	0.078
	Medical supplement	Vitamin D	16.0%	4.9%	0.019 *
		Iron	11.3%	9.9%	0.815
		Vitamin complex	15.1%	4.9%	0.031 *
	Sport Performance	Caffeine	31.1%	32.1%	1.000
		Creatine	21.7%	9.9%	0.046 *
Group B		Vitamin C	17.9%	8.6%	0.088
		BCAA	17.0%	8.6%	0.129
		L-Carnitine	7.5%	13.6%	0.223
Group C		Green Tea	12.3%	11.1%	1.000
		Royal Jelly	10.4%	12.3%	0.816
		Glutamine	13.2%	7.4%	0.239

Data are frequencies for 112 men and 75 women (106 professional players and 81 amateur players). * Statistical difference in the frequency of consumption between groups (*p* < 0.05). Group A: Supplements with strong scientific evidence for use in specific situations in sport using evidence-based protocols; Group B: supplements with emerging scientific support, deserving of further research; Group C: supplements with scientific evidence not supportive of benefit amongst athletes. BCAA: Branched Chain Amino Acids.
